# Otitis Media and Its Association With Hearing Loss in Chinese Adults: A Population Based Study of 4 Provinces in China

**DOI:** 10.3389/fpubh.2022.852556

**Published:** 2022-05-16

**Authors:** Yanan Luo, Ping He, Xu Wen, Rui Gong, Xiangyang Hu, Xiaoying Zheng

**Affiliations:** ^1^Department of Global Health, School of Public Health, Peking University, Beijing, China; ^2^China Center for Health Development Studies, Peking University, Beijing, China; ^3^College of Biochemical Engineering, Beijing Union University, Beijing, China; ^4^China Rehabilitation Research Center for Deaf Children, Beijing, China; ^5^Chinese Academy of Medical Sciences/Peking Union Medical College, School of Population Medicine and Public Health, Beijing, China

**Keywords:** otitis media, hearing loss, rural-urban disparities, adults, China

## Abstract

**Objective:**

Otitis media is a recognized cause of significant hearing loss, particularly in developing countries. This study aimed to investigate the relationship between otitis media and hearing loss in Chinese adults aged 18 years and older.

**Methods:**

The survey was based on WHO Ear and Hearing Disorders Survey Protocol and 36,783 adults at the ages between 18 years and above were selected in this study. Trained local examiners performed pure tone audiometry to screen people with hearing loss, and those who were screened positively for hearing loss were referred to audiologists to make final diagnosis. All participants underwent clinical ENT check-up and otoscopic examination by doctors trained in ENT. Each participant was assigned a single middle ear diagnosis. Diagnoses were assigned as per the WHO classification of ear and hearing disorders.

**Results:**

Logistic regressions showed that higher prevalence of hearing loss was found in participants with otitis media, with an unadjusted odds ratio of 5.67 (*95%CI*: 4.66, 6.90). The next two models (Model 2–3) had slight impact on ORs. The interaction of residency and otitis media was statistically significant (*OR* = 1.70, *95%CI* = 1.15, 2.53); otitis media patients in rural areas had higher risk of hearing loss. However, this interaction became not significant in 65 years old and above participants.

**Conclusions:**

Otitis media was associated with the risk of hearing loss. Compared with urban patients with otitis media, rural patients have the higher risk of hearing loss. Action to reduce the risk of hearing loss in otitis media will require attention to rural-urban disparities.

## Introduction

As one of the most common sensory impairments, hearing loss has become a public health concern across the globe (1). According to the report, around 5.3% of the world population suffered from disabling hearing loss and 90% of them were adults in 2012 (2). Hearing loss contributes a high burden to patients, family members, healthcare system and long-term care system, which leads to communication difficulties, impaired cognitive functioning and reduced quality of life (3). Global Burden of Disease Study 2013 showed that hearing loss was ranked as the fifth top cause of years lived with disability, which was higher than other chronic diseases including diabetes, dementia and chronic obstructive pulmonary disease (1, 4). In China, about 11% of adults suffered from disabling hearing loss according to the Second Sample Survey on Disability in 2006 (5).

Otitis media (OM) defined as inflammation involving the mucosal lining of middle ear cleft, which includes acute otitis media (AOM), otitis media with effusion (OME) and chronic suppurative otitis media (CSOM) (6). The complications and sequelae of OM are important causes of preventable hearing loss, contributing a high burden to healthcare system (7). The complications of OM lead to 28 thousand deaths each year according to WHO's report (8). Among different varieties of OMs, AOM has rare intralabyrinthine complications leading to deafness, and brings a substantial burden of hearing loss and suppurative complications, which results in excessive antibiotic consumption in most countries (9). OME has high relapse rate and imposes a great health care burden. After an initial episode of OME, the recurrence rate of OME can be as high as 40% (10). In 2008, 100 to 400 million Australian Dollars spent on the treatment for both AOM and OM (10). CSOM is an important cause of preventable hearing loss, particularly in developing countries (7).

OM is a significant cause of preventable hearing loss, particularly in developing countries (11). This relationship between OM and hearing loss has attracted increased attention before. Evidence from Nijeria observed that OM attributed to 25.8% of preventable hearing loss (11). The complications and sequelae of different kinds of middle ear diseases, including CSOM, OME and AOM, were associated with hearing loss (9, 12, 13). The prevalence of hearing loss in CSOM (14), OME (15) and AOM (11) groups is 60, 18.4, and 9.1%, respectively. Up to now, few reports examined the distribution of OM presenting with hearing loss, and the association between OM and hearing loss in Chinese mainland.

Using a cross-sectional, population-based Ear and Hearing Disorder Survey in four provinces of China, this study aimed to investigate the relationship between OM and hearing loss in Chinese adults aged 18 years and older. A better knowledge of the prevalence of AOM, CSOM, OME and their realtionships with hearing loss will fill the gaps on this topic in China, which is necessary to adequately assess the demand of interventions for hearing loss, and form the basis of a public health program in China, as well as provide data for the WHO Prevention for Blindness and Deafness 2020.

## Methods

### Participants

We obtained data from Ear and Hearing Disorder Survey, which was a population-based study conducted in four provinces of China (Jilin, Guangdong, Gansu, and Shaanxi) between August 2014 and September 2015. The survey design was established by a technical team based on WHO Ear and Hearing Disorder Survey Protocol (16), which has been used in China (17, 18). The screening scale of audiometry was on the basis of the modified version of the WHO/PBD Ear and Hearing Disorders Examination Form (Version 8.3) (19).

**Figure 1:**
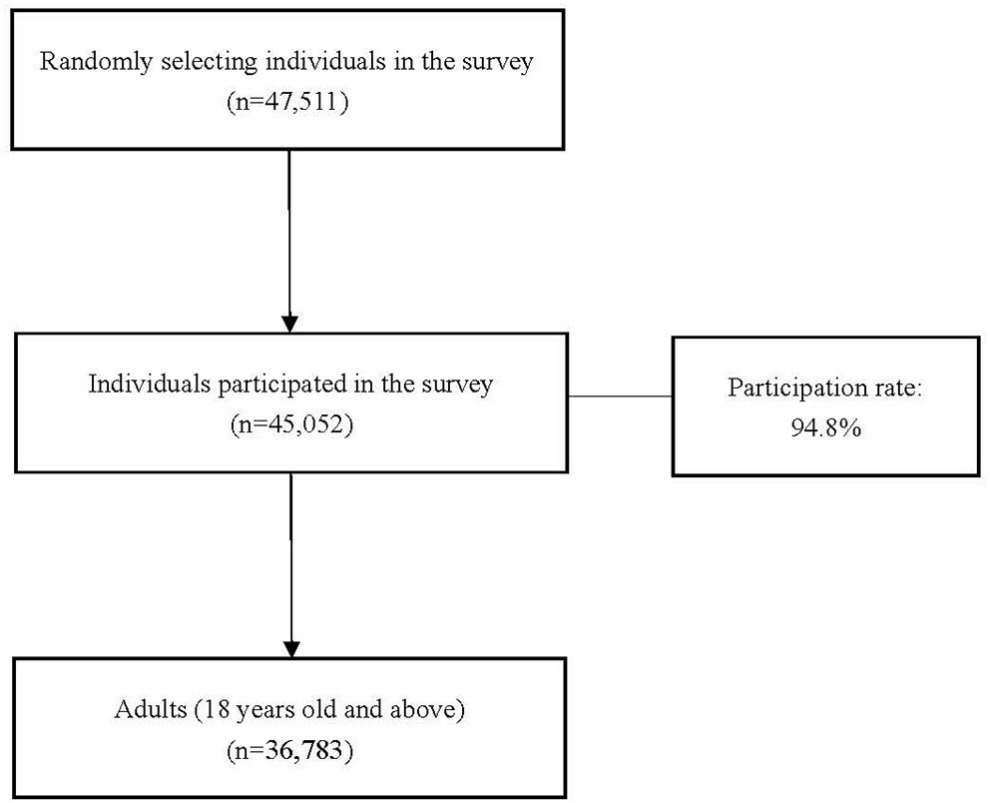
Flowchart of study samples.

The sampling frame covered almost 200 million people, representing about one in seven of the total population in China. Probability proportion to size (PPS) sampling method was used to identify 144 sites from 24 counties or districts in four provinces. Each site included 100 households which had lived in the registered address for over 6 months. A total of 47,511 individuals were randomly selected and 45,052 of them participated in the survey, yielding a participation rate of 94.80% (17). All participants consented to participate in the survey, and if required, to be subsequently examined by audiologists. In this study, we restricted our analysis to 36,783 adults at the ages between 18 years and above. The flowchart of the study samples could be found in Figure 1.

### Diagnostic Assessment

Audiometry was performed by trained examiners according to the established protocols of Ear and Hearing Disorder Survey. Examiners were recruited from local primary health care institutions (village doctors in rural area and community physicians in urban area), and were trained by provisional technical teams in survey skills and audiological techniques. Before the household survey, examiners went to local sites to select audiometric test rooms with ambient noise levels not exceeding 40 dBA measured by sound level meters, and were equipped with MADSEN-Xeta pure tone audiometers and other required examination equipment. During the survey, noise-excluding headsets were used if the ambient noise of testing rooms exceeded 40 dB. Audiometric equipment was calibrated by a laboratory at the beginning and end if a study, and on a daily basis by team members using self-calibration against their known hearing levels. During and after the survey, a subsample with 5% of participants were rechecked and the consistency rates of rechecks both reached 90% and above (17).

Examiners performed pure tone audiometry among people aged 18 years old and above in a selected quiet room. Both ears were tested separately at 0.5, 1, 2, and 4 kHz to obtain the hearing threshold on each frequency point. In this survey, each participant in our study has at least one ear, and all of them were tested in one or both ears. Pure tone threshold averages in the better ear were calculated to identify hearing loss and its grades. According to the standard of WHO/PDH/97.3 (16), hearing loss was defined as pure-tone average >25 dB in better ear, and the categories were collapsed to simplify analyses: normal hearing ≤ 25 dB, mild hearing loss >25 and ≤ 40 dB, moderate hearing loss >40 and ≤ 60 dB and severe hearing loss >60 dB. If individuals who cannot respond reliably in pure tone audiometry and when hearing loss is suspected, audiologists would use auditory brain stem response (ABR) and otoacoustic emission (OAE) to do the further diagnosis.

All participants underwent clinical ENT check-up and otoscopic examination by doctors trained in ENT. A normal diagnosis was assigned if participants had healthy middle ear cleft bilaterally. If patients had a differing diagnosis in each ear, the most clinically serious diagnosis was taken: CSOM, followed by OME and AOM. Of these, AOM is diagnosed among patients with acute onset, presence of middle ear effusion, physical evidence of middle ear inflammation or of fever symptoms.

### Ethics Approval

This survey was ethically approved by China Disabled Persons' Federation (number: 2014&ZZ028). The committee board, which was composed of audiologists and epidemiologists, reviewed the study protocols and ethical situations. The authors had no access to identifying information for the study participants prior to data analysis. All participants signed the informed consent with interviewers to participate in the survey and clinical diagnosis. For those with severe hearing loss who were unable to sign the consent, family members represented them to sign the consent.

### Measures

The outcome variable was whether or not an adult had hearing loss. The independent variable was whether or not an adult had OM. Covariates included socioeconomic status, defined by three categorical variables: occupation (white-collar worker, farming worker, blue-collar worker, others and the unemployed), education (illiteracy, primary school, junior high school and senior high school or above) and income (tertiles of annual family income per capita, tertile 1 represented those with <963$ annual income, tertile 2 ~ 963$ to 2,087$ annual income, and tertile 3 ~ >2,087$). White-collar workers in this study involved professional and governmental employees, and blue-collar workers referred to manual and services-oriented workers excluding farmers (20). Covariates included sex (male and female), having spouse (yes and no) and age (year, continuous variable). The covariates were all self-reported. AOM, OME and CSOM were all dummy variables.

### Analytical Approach

Descriptive statistics were used to present the proportion of hearing loss by various demographic characteristics and OM. Fisher exact test was conducted to compare the differences of the characteristics. Multivariate logistic regression models allowing for multiple demographic and socioeconomic covariates and stepwise approach were used to evaluate the association between OM and hearing loss. The odds ratios (ORs) with 95% confidence intervals (CIs) were presented. A P value less than 0.05 was considered statistically significant. χ^2^ test was used to examine the difference on prevalence of people with hearing loss by OM. The software Stata version 13.0 for Windows (Stata Corp., College Station, TX, USA) was utilized for statistical analysis. Because location is also one of the independent variable that may affect both status of OM and hearing loss, all analyses were conducted separately by urban and rural areas.

## Results

### Characteristics of Participants in Chinese Adults

In our study, out of 36,783 adults in the survey, there were 307 (0.83%) with OM and 7,360 (20.01%) had hearing loss. 0.83% adults had OM and 20.01% suffered from hearing loss. Among patients with OM, CSOM accounted for 89.44%, OM with effusion with 9.32%, and acute OM 0.62%.

In patients with hearing loss, most were mild (68.72%), and moderate and severe hearing losses accounted for 21.62 and 9.66%, respectively. Compared with participants without hearing loss (OM, 0.35%), more hearing loss patients had OM (2.77%). Older people, more males, more illiteracy and the lowest income per capita groups were in hearing loss groups. More details are presented in Table 1.

**Table 1:** Characteristics of participants, *N* (% or Mean).

**Variable**	**Hearing Loss**					***P***
	**Yes**, ***n*** **(%)/mean (SD)**				**No, *n* (%)**	
	**Mild**	**Moderate**	**Severe**	**Total**		
Total	5,058 (68.72)	1,591 (21.62)	711 (9.66)	7,360 (20.01)	29,423 (79.99)	/
Otitis media						<0.001
No otitis media	4,963 (98.12)	1,530 (96.17)	663 (93.25)	7,156 (97.23)	29,320 (99.65)	
Any otitis media	95 (1.88)	61 (3.83)	48 (6.75)	204 (2.77)	103 (0.35)	
Age, years (mean, SD)	57.98 (12.52)	66.48 (12.67)	66.02 (15.51)	60.60 (13.44)	40.74 (13.59)	
Age group						<0.001
18–64	3,473 (68.66)	634 (39.85)	284 (39.94)	4,391 (59.44)	27,971 (95.07)	
65+	1,585 (31.34)	957 (60.15)	427 (60.06)	2,996 (40.56)	1,452 (4.93)	
Sex						<0.001
Male	2,607 (51.54)	830 (52.17)	402 (56.54)	3,839 (52.16)	14,304 (48.62)	
Female	2,451 (48.46)	761 (47.83)	309 (43.46)	3,521 (47.84)	15,119 (51.38)	
Residence						0.865
Urban	2,634 (52.08)	779 (48.96)	306 (43.04)	3,719 (50.53)	14,833 (50.41)	
Rural	2,424 (47.92)	812 (51.04)	405 (56.96)	3,641 (49.47)	14,590 (49.59)	
Having spouse						<0.001
No	727 (14.39)	388 (24.43)	221 (31.21)	1,336 (18.18)	6,006 (20.44)	
Yes	4,324 (85.61)	1,200 (75.57)	487 (68.79)	6,011 (81.82)	23,382 (79.56)	
Missing						
Education						<0.001
Illiteracy	2,118 (41.87)	934 (58.71)	466 (65.54)	3,518 (47.8)	6,023 (20.47)	
Primary school	1,658 (32.78)	368 (23.13)	145 (20.39)	2,171 (29.5)	10,521 (35.76)	
Junior high school and above	1,276 (25.23)	285 (17.91)	98 (13.78)	1,659 (22.54)	12,849 (43.67)	
Missing	6 (0.12)	4 (0.25)	2 (0.28)	12 (0.16)	30 (0.10)	
Income per capital^a^						<0.001
Tertile 1	1,539 (30.43)	609 (38.28)	315 (44.3)	2,463 (33.46)	9,033 (30.70)	
Tertile 2	1,609 (31.81)	451 (28.35)	234 (32.91)	2,294 (31.17)	9,829 (33.41)	
Tertile 3	1,910 (37.76)	531 (33.38)	162 (22.78)	2,603 (35.37)	10,559 (35.89)	
Missing	0 (0.00)	0 (0.00)	0 (0.00)	0 (0.00)	2 (0.01)	

a *Tertile 1 represents those with <963$ annual income, tertile 2 ~ 963$ to 2,087$ annual income, and tertile 3 ~ >2,087$*.

### Prevalence of Hearing Loss by OM in Chinese Adults

**Figure 2:**
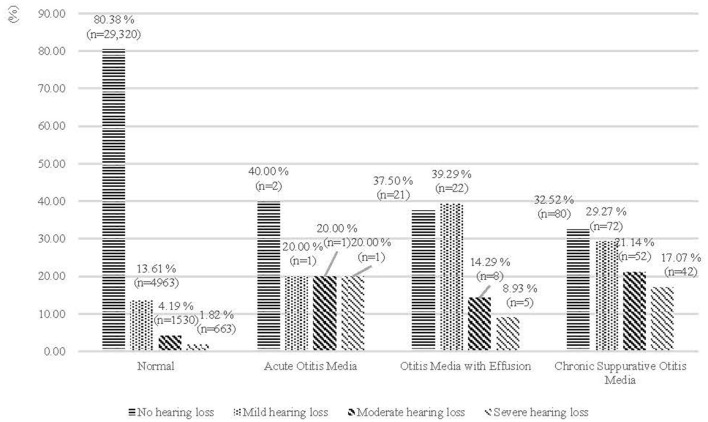
Prevalence of hearing loss in Chinese adults (%), by otitis media (n of total population = 36,783).

**Figure 3:**
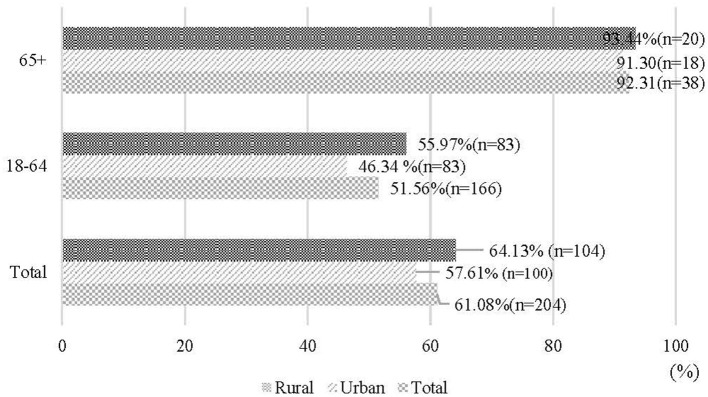
Prevalence of hearing loss in patients with otitis media (%), by age group (n of otitis media patients = 204).

Figure 2 presents the distribution of Chinese adult participants with hearing loss according to presence of OM. Out of those with OM, the prevalence of mild, moderate and severe hearing losses were 29.44, 18.78, and 12.86%, respectively. Figure 3 shows that patients with OM living in rural areas had a higher prevalence of hearing loss (64.13%) than those living in urban areas (57.13%) (*P* < 0.001), similarly occurring in the 18–64 age group, with 55.97% for rural areas, and 46.34% for urban areas. However, for those 65 years old and above, the rates are almost similar with 91.30 and 93.44% in rural and urban areas, respectively.

### Association Between OM and Hearing Loss

Table 2 presents the results of logistic regression between OM and hearing loss. Odds ratios in multivariate analyses virtually confirmed the association between OM and hearing loss. Higher prevalence of hearing loss was found in participants with OM, with the adjusted odds ratio of 5.67 (*95%CI*: 4.66, 6.90) (Table 2). The interaction of residency and OM was statistically significant (*OR* = 1.70, *95%CI* = 1.15, 2.53); OM patients in rural areas had higher risk of hearing loss (Table 3). This interaction remained significant in adults aged 18–64 years old (Table 4). However, it became not significant in 65 years old and above participants (Table 5).

**Table 2:** Logistic regression analysis: 18 years old and above (main effects).

**Variables**	**B**	**SE**	***P*** **-value**	**Exp (B)**	**95%CI for exp (B)**
Otitis media (Reference = No)	2.09	0.14	<0.001	5.67	4.66–6.90
Residency (Reference = Urban)	–.26	0.04	<0.001	0.90	0.85–0.96
Age, years	0.10	0.01	<0.001	1.11	1.10–1.11
Sex (Reference = Male)	−0.30	0.03	<0.001	0.77	0.72–0.81
Having spouse (Reference = Yes)	−0.15	0.05	0.002	0.86	0.78–0.94
Education (Reference = Illiteracy)				
Primary school	−0.11	0.04	0.010	0.89	0.83–0.97
Junior high school and above	−0.45	0.05	<0.001	0.64	0.58–0.70
Income per capital^a^ (Reference = tertile 1)				
Tertile 2	0.01	0.04	0.856	1.01	0.93–1.09
Tertile 3	0.09	0.04	0.046	1.09	1.00–1.19
*R* ^2^	0.29				

a *Tertile 1 represents those with <963$ annual income, tertile 2 ~ 963$ to 2,087$ annual income, and tertile 3 ~ >2,087$*.

**Table 3:** Logistic regression analysis: 18 years old and above (Main effects and interaction effects).

**Variables**	**B**	**SE**	**P-value**	**Exp (B)**	**95%CI for exp (B)**
**Main effects**			
Otitis media (Reference = No)	1.93	0.21	<0.001	4.24	3.18–5.66
Residency (Reference = Urban)	−0.26	0.04	<0.001	0.76	0.70–0.82
Age, years	0.10	0.01	<0.001	1.10	1.10–1.10
Sex (Reference = Male)	−0.30	0.03	<0.001	0.74	0.69–0.79
Having spouse(Reference = Yes)	−0.15	0.05	0.002	0.86	0.79–0.95
Education(Reference = Illiteracy)			
Primary school	−0.11	0.04	0.006	0.89	0.83–0.97
Junior high school and above	−0.45	0.05	<0.001	0.64	0.58–0.70
Income per capital^a^ (Reference = tertile 1)				
Tertile 2	0.01	0.04	0.846	1.01	0.93–1.09
Tertile 3	0.09	0.04	0.046	1.09	1.00–1.19
**Interaction effects**				
Residency × Otitis media (Reference = urban × Otitis media)	0.29	0.28	0.301	1.70	1.15–2.53
*R* ^2^	0.29				

a *Tertile 1 represents those with <963$ annual income, tertile 2 ~ 963$ to 2,087$ annual income, and tertile 3 ~ >2,087$*.

**Table 4:** Logistic regression analysis: 18–64 years old (Main effects and interaction effects).

**Variables**	**B**	**SE**	***P*-value**	**Exp (B)**	**95%CI for exp (B)**
**Main effects**					
Otitis media (Reference = No)	1.99	0.22	<0.001	4.25	3.12–5.79
Residency (Reference = Urban)	−0.27	0.04	<0.001	0.74	0.68–0.81
Age, years	0.10	0.00	<0.001	1.10	1.10–1.11
Sex (Reference = Male)	−0.33	0.04	<0.001	0.72	0.67–0.77
Having spouse(Reference = Yes)	−0.21	0.06	<0.001	0.81	0.72–0.91
Education(Reference = Illiteracy)				
Primary school	−0.14	0.05	0.003	0.87	0.79–0.95
Junior high school and above	−0.49	0.06	<0.001	0.61	0.55–0.69
Income per capital^a^ (Reference = tertile 1)					
Tertile 2	−0.03	0.05	0.567	0.97	0.89–1.07
Tertile 3	0.03	0.05	0.612	1.03	0.93–1.13
**Interaction effects**				
Residency × Otitis media (Reference = urban × Otitis media)	0.19	0.30	0.514	1.67	1.10–2.54
*R* ^2^	0.19				

a *Tertile 1 represents those with <963$ annual income, tertile 2 ~ 963$ to 2,087$ annual income, and tertile 3 ~ >2,087$*.

**Table 5:** Logistic regression analysis: 65 years old and above (Main effects and interaction effects).

**Variables**	**B**	**SE**	***P*** **-value**	**Exp (B)**	**95%CI for exp (B)**
**Main effects**				
Otitis media (Reference = No)	1.33	0.62	0.031	4.41	1.89–10.31
Residency (Reference = Urban)	−0.17	0.08	0.044	0.84	0.72–1.00
Age, years	0.07	0.01		1.07	1.06–1.08
Sex (Reference = Male)	−0.19	0.07	0.007	0.83	0.72–0.95
Having spouse (Reference = Yes)	−0.17	0.08	0.040	0.84	0.72–1.00
Education (Reference = Illiteracy)			
Primary school	−0.05	0.09	0.624	0.95	0.80–1.14
Junior high school and above	−0.22	0.11	0.050	0.80	0.64–1.00
Income per capital^a^ (Reference = tertile 1)			
Tertile 2	0.11	0.08	0.202	1.11	0.94–1.31
Tertile 3	0.30	0.09	0.002	1.35	1.12–1.63
**Interaction effects**			
Residency × Otitis media (Reference = urban × Otitis media)	1.45	1.19	0.226	1.93	0.51–7.29
*R* ^2^	0.039				

a *Tertile 1 represents those with <963$ annual income, tertile 2 ~ 963$ to 2,087$ annual income, and tertile 3 ~ >2,087$*.

## Discussion

The association of significant hearing loss with OM is well-documented in previous literature, and its auditory consequences also recognized (21). However, there is little evidence on the association between OM and hearing loss in Chinese mainland adults due to the data resources limited before. This is the first population-based study on the association between OM and hearing loss in Chinese mainland adults. According to the WHO Ear and Hearing Disorders Survey Protocol, individuals were first screened by local trained examiners using pure tone audiometry and were further referred to audiologists for final diagnosis of hearing loss. Using a population-based Ear and Hearing Disorder Survey in four provinces of China, this study showed that OM was associated with the risk of hearing loss. Compared with urban patients with OM, rural patients have the higher risk of hearing loss.

OM is a major common risk of hearing loss. Possible explanation of hearing loss associated with OM have focused on the routes between middle and inner ear (22). Firstly, the inflammatory mediators of middle ear effusion might trigger immune responses within the cochlea, leading to sensorineural hearing loss (23). Secondly, alteration of the ossicular chain resulting from middle ear effusion or mucosal edema within the tympanic space would contribute to hearing loss (24). Thirdly, the rupture of the tympanic membrane and the consequences of chronic inflammation from OM may result in conductive hearing loss (25).

Our finding showed that the prevalence of hearing loss caused by OM was around 60% in Chinese adults, and this prevalence varied across different types of OM and increased with age. Evidence from Nigeria and Denmark reported that more than half of OM patients experienced hearing loss, which was similar to our results (9, 26). Evidence also indicated that the risk of hearing loss varied across the duration and types of OMs (27). Among all types of OMs, CSOM is a major cause of hearing loss in many developing countries. WHO's estimates that 65 million to 330 million individuals worldwide develop CSOM, 60% of whom will suffer from hearing impairment (9). After considered with OME, OM was evidenced that accounted for more than 75% of hearing loss (26). Furthermore, similar patterns of hearing loss prevalence and age was found in previous studies, with the prevalence of OM related hearing loss in the age group 65–74 was five times the prevalence in the first year of life (7).

Our results showed that the association between OM and the risk of hearing impairment was stronger in residents of rural areas than indiviudals in urban areas. Geographical distribution of hearing loss due to OM is uneven, and socioecnomic conditions may account for a major part of the difference (9, 28). Compared with urban residents, rural dewellers in China have lower level of education and are usually short of sufficient medical knowledge. And the health literacy between rural and urban residents may contribute to such differences. Lack of medical knowledge may become an important obstacle against a broader involvement of ear diseases therapy and hearing loss prevention and intervention (29). The deficiency of adequate treatment for ear diseases may delay their recovery and increase the risk of hearing loss. Further, hearing loss is very easy to be neglected because there is usually no pain or discomfort associated with symptoms, especially for rural residents with low level of medical knowledge. This may lead rural inhabitants to be affected by a more severe form of hearing loss from OM. Moreover, compared with the patients in urban areas, rural areas patients have disadvantaged conditions in accessing to diagnosis and treatment of ear diseases, unfavorable sanitation conditions and expensive expenditure due to the incompleted health insurance and health care systems in rural China (30).

## Limitations

This study has several limitations. First, due to the restriction of our study design, we did not include the exposure duration of OMs, which may affect the response variability. For example, OMs patients in rural areas always have a longer duration exposure to OMs than urban patients, which may widen the urban-rural disparities of OMs related hearing loss. Therefore, we need caution in interpreting our results. Furthermore, a cross-sectional study design cannot draw causal inferences. From this perspective, further studies are necessary to investigate the causality and its pathways between OM and hearing loss drawing from prospective cohort design in Chinese mainland.

Despite these limitations, the strengths of this study include a large-size, population-based design in four provinces of China based on the WHO Ear and Hearing Disorder Survey Protocol, as well as hearing conditions ascertained by audiologists according to the WHO criteria. It is the first time to study the urban-rural disparity of the association between OMs and hearing loss in Chinese adults.

## Conclusions

This study investigated the association between OMs and hearing loss among Chinese adults based on a population-based study conducted in four provinces of China between August 2014 and September 2015 by using a multivariate analysis with logistic regression. Our results showed that among OM patients, 29.44, 18.78, and 12.86% was the prevalence of mild, moderate and severe hearing loss, respectively. We found that OM was associated with the risk of hearing loss. Compared with urban patients with OM, rural patients have the higher risk of hearing loss. This study contributes to the literature on ear disease and hearing loss in developing nations of a non-Western context. Action to reduce the risk of hearing loss in OM will require attention to rural-urban disparities.

## Data Availability Statement

The datasets presented in this article are not readily available because the acquisition of the dataset requires the consent of China Rehabilitation Research Center for Deaf Children, which is incharege of the date management. Requests to access the datasets should be directed to webmaster@crrchsi.org.cn; http://www.chinadeaf.org.

## Ethics Statement

This survey was ethically approved by China Disabled Persons' Federation (number: 2014 & ZZ028). The patients/participants provided their written informed consent to participate in this study.

## Author Contributions

YL, PH, and XW performed conceived and designed the study, data analysis, and wrote the manuscript. RG and XH participated in conducting the study and supervised data collection. XZ supervised all aspects of its implementation and contributed to writing the article. All authors contributed to the article and approved the submitted version.

## Funding

This work was supported by Major Project of the National Social Science Fund of China (21&ZD187), National Social Science Fund of China (21CRK014), Promotion of Initiative and Empowerment for Women (ANSO-SBA-2020-02), Beijing Social Science Foundation (Grant No. 20SRC029), and Beijing Union University Foundation (Grant No. BPHR2020DS05). The funders had no role in study design, data collection and analysis, decision to publish and preparation of the manuscript.

## Conflict of Interest

The authors declare that the research was conducted in the absence of any commercial or financial relationships that could be construed as a potential conflict of interest.

## Publisher's Note

All claims expressed in this article are solely those of the authors and do not necessarily represent those of their affiliated organizations, or those of the publisher, the editors and the reviewers. Any product that may be evaluated in this article, or claim that may be made by its manufacturer, is not guaranteed or endorsed by the publisher.

## References

[1] Looi LM Ganten D McGrath PF Gross M Griffin GE. Hearing loss: a global health issue. Lancet. (2015) 385:943–4. 10.1016/S0140-6736(15)60208-2 25743174

[2] Hearing loss: an important global health concern. Lancet. (2016) 387:2351. 10.1016/S0140-6736(16)30777-2 27312288

[3] Dalton DS Cruickshanks KJ Klein BE Klein R Wiley TL Nondahl DM. The impact of hearing loss on quality of life in older adults. Gerontologist. (2003) 43:661–8. 10.1093/geront/43.5.661 14570962

[4] Global regional and national incidence prevalence and years lived with disability for 301 acute and chronic diseases and injuries in 188 countries 1990–2013: 1990–2013: a systematic analysis for the Global Burden of Disease Study 2013. Lancet. (2015) 386:743–800. 10.1016/s0140-6736(15)60692-4 26063472 PMC4561509

[5] Zheng X Chen G Song X Liu J Yan L Du W. Twenty-year trends in the prevalence of disability in China. Bull World Health Organiz. (2011) 89:788–97. 10.2471/BLT.11.089730 22084524 PMC3209727

[6] Bluestone CD Tunkel DE Grundfast KM. Pediatric otolaryngology. Pediatric clinics of North America. (2003) 50.

[7] Monasta L Ronfani L Marchetti F Montico M Vecchi Brumatti L Bavcar A. Burden of disease caused by otitis media: systematic review and global estimates. PLoS ONE. (2012) 7:e36226. 10.1371/journal.pone.0036226 22558393 PMC3340347

[8] Acuin J. Chronic Suppurative Otitis Media: Burden of Illness and Management Options. Geneva: World Health Organization (2004).

[9] Vergison A Dagan R Arguedas A Bonhoeffer J Cohen R Dhooge I. Otitis media and its consequences: beyond the earache. Lancet Infect Dis. (2010) 10:195–203. 10.1016/S1473-3099(10)70012-8 20185098

[10] Cai T McPherson B. Hearing loss in children with otitis media with effusion: a systematic review. Int J Audiol. (2017) 56:65–76. 10.1080/14992027.2016.1250960 27841699

[11] Li N Chen G Song X Du W Zheng X. Prevalence of autism-caused disability among Chinese children: a national population-based survey. Epilepsy Behav. (2011) 22:786–9. 10.1016/j.yebeh.2011.10.002 22079437

[12] Blakley BW Kim S. Does chronic otitis media cause sensorineural hearing loss? J Otolaryngol. (1998) 27:17–20. 9511114

[13] Li N Chen G Du W Song X Zhang L Zheng X. Population-level prevalence estimate and characteristics of psychiatric disability among Chinese adults. J Psychiatr Res. (2011) 45:1530–4. 10.1016/j.jpsychires.2011.07.001 21794875

[14] Hastert TA Beresford SA Sheppard L White E. Disparities in cancer incidence and mortality by area-level socioeconomic status: a multilevel analysis. J Epidemiol Community Health. (2015) 69:168–76. 10.1136/jech-2014-204417 25288143

[15] Boudewyns A Declau F Van den Ende J Van Kerschaver E Dirckx S Hofkens-Van den Brandt A. Otitis media with effusion: an underestimated cause of hearing loss in infants. Otol Neurotol. (2011) 32:799–804. 10.1097/MAO.0b013e31821b0d07 21593700

[16] World Health Organization. WHO Ear and Hearing Disorders Survey Protocol for a Population-Based Survey of Prevalence and Causes of Deafness and Hearing Impairment and Other Ear Disorders. Geneva: World Health Organization (1999).

[17] Hu XY Zheng XY Ma FR Long M Han R Zhou LJ. [Prevalence of hearing disorders in China: a population-based survey in four provinces of China]. Zhonghua er bi Yan Hou Tou Jing Wai ke za zhi. (2016) 51:819–25. 10.3760/cma.j.issn.1673-0860.2016.11.004 27938607

[18] Bu X Liu C Xing G Zhou L Liang C Zheng Y. WHO Ear and Hearing Disorders Survey in four provinces in China. Audiological Medicine. (2011) 9:141–6. 10.3109/1651386X.2011.63128527938607

[19] World Health Organization. WHO/PBD Ear and Hearing Disorders Examination Form Version 8.3. (2012). Available online at: http://www.who.int/blindness/Ear_hearingsurveyformupdtaed.pdf (accessed May 7, 2017).

[20] Wang H Ma L Yin Q Zhang X Zhang C. Prevalence of alcoholic liver disease and its association with socioeconomic status in north-eastern China. Alcohol Clin Exp Res. (2014) 38:1035–41. 10.1111/acer.12321 24428769

[21] Olusesi AD. Otitis media as a cause of significant hearing loss among Nigerians. Int J Pediatr Otorhinolaryngol. (2008) 72:787–92. 10.1016/j.ijporl.2008.02.008 18378007

[22] Li N Zhang L Du W Pang L Guo C Chen G. Prevalence of dementia-associated disability among Chinese older adults: results from a national sample survey. Am J Geriatr Psychiatry. (2015) 23:320–5. 10.1016/j.jagp.2014.06.002 25488495

[23] Dobie RA Berlin CI. Influence of otitis media on hearing and development. Ann Otol Rhinol Laryngol Suppl. (1979) 88(5 Pt 2 Suppl. 60):48–53. 10.1177/00034894790880S505 115362

[24] Daly KA Pirie PL Rhodes KL Hunter LL Davey CS. Early otitis media among Minnesota American Indians: the Little Ears Study. Am J Public Health. (2007) 97:317–22. 10.2105/AJPH.2004.052837 17194873 PMC1781377

[25] Elemraid MA Brabin BJ Fraser WD Harper G Faragher B Atef Z. Characteristics of hearing impairment in Yemeni children with chronic suppurative otitis media: a case-control study. Int J Pediatr Otorhinolaryngol. (2010) 74:283–6. 10.1016/j.ijporl.2009.12.004 20042241

[26] Jensen RG Koch A Homøe P. The risk of hearing loss in a population with a high prevalence of chronic suppurative otitis media. Int J Pediatr Otorhinolaryngol. (2013) 77:1530–5. 10.1016/j.ijporl.2013.06.025 23906989

[27] Singer AEA Abdel-Naby Awad OG El-Kader RMA Mohamed AR. Risk factors of sensorineural hearing loss in patients with unilateral safe chronic suppurative otitis media. Am J Otolaryngol. (2018) 39:88–93. 10.1016/j.amjoto.2018.01.002 29331307

[28] Shaheen MM Nahar S. Comparison of chronic suppurative otitis media in rural and urban primary school children in Bangladesh. J Laryngol Otol. (2014) 128:499–503. 10.1017/S0022215114001054 24895917

[29] Kasliwal N Joshi S Pareek SM. Determinants of sensorineural hearing loss in chronic middle-ear disease. Indian J Otolaryngol Head Neck Surg. (2004) 56:269. 10.1007/BF02974385 23120094 PMC3451147

[30] Wang J Chen B Xu M Wu J Wang T Zhao J. Etiological factors associated with chronic suppurative otitis media in a population of Han adults in China. Acta Oto Laryngol. (2016) 136:1024–8. 10.1080/00016489.2016.1183818 27206699

